# Lupus anticoagulant hypoprothrombinemia syndrome associated with bilateral adrenal haemorrhage in a child: early diagnosis and intervention

**DOI:** 10.1186/s12959-021-00271-0

**Published:** 2021-03-17

**Authors:** Atsushi Sakamoto, Masao Ogura, Atsushi Hattori, Kinji Tada, Reiko Horikawa, Hisaya Nakadate, Kimikazu Matsumoto, Keiji Nogami, Masahiro Ieko, Akira Ishiguro

**Affiliations:** 1 Center for Postgraduate Education and Training, National Center for Child Health and Development, NCCHD, Tokyo, Japan; 2 Children’s Cancer Center, National Center for Child Health and Development, Setagaya-ku, Tokyo, 157-8535 Japan; 3 Division of Nephrology and Rheumatology, NCCHD, Tokyo, Japan; 4 Division of Endocrinology and Metabolism, NCCHD, Tokyo, Japan; 5 Division of Hematology, NCCHD, Tokyo, Japan; 6 Department of Pediatrics, Nara Medical University, Kashihara, Nara, Japan; 7 Department of Internal Medicine, School of Dentistry, Health Sciences University of Hokkaido, Ishikari-Tobetsu, Hokkaido Japan

**Keywords:** Lupus anticoagulant, Hypoprothrombinemia, Adrenal haemorrhage, Adrenal insufficiency, Lupus anticoagulant hypoprothrombinemia syndrome

## Abstract

**Background:**

Lupus anticoagulant-hypoprothrombinemia syndrome (LAHPS) is characterized by bleeding and thrombosis in patients with autoimmune diseases or infections. Paediatric LAHPS exhibits various degrees of bleeding, ranging from mild to severe; however, adrenal haemorrhage due to LAHPS and its long-term clinical course have not been sufficiently described.

**Case presentation:**

A 9-year-old boy presented with prolonged abdominal pain and abnormal coagulation screening tests. The laboratory tests showed prolonged activated partial thromboplastin time and subsequently revealed the presence of lupus anticoagulant, anti-nuclear antibodies, and hypoprothrombinemia, leading to diagnosis of LAHPS. An enhanced computed tomogram demonstrated nodular lesions in the adrenal glands bilaterally, suggestive of adrenal haemorrhage. Laboratory and clinical manifestations exhibited life-threatening adrenal insufficiency that required hydrocortisone administration. The patient developed systemic lupus erythematosus, diagnosed 12 months later.

**Conclusions:**

This patient with LAHPS developed rare adrenal failure due to adrenal haemorrhage, a life-threatening event that should be recognized and treated early. In our case, renal dysfunction was also observed when systemic lupus erythematosus was diagnosed 1 year after LAHPS. Our case emphasizes that early recognition of adrenal failure and careful long-term observation is required in patients with autoantibodies.

## Background

Lupus anticoagulant (LA) is an antiphospholipid antibody that inhibits phospholipid-dependent clotting without inhibiting the activity of individual coagulation factors, and often leads to severe thrombotic disorders [1]. Patients with concomitant acquired hypoprothrombinemia and LA, termed lupus anticoagulant-hypoprothrombinemia syndrome (LAHPS), sometimes show decreased coagulation factor activity [2, 3]. Patients occasionally develop LAHPS after viral infections, and present with bleeding symptoms in the paediatric age group [2]. Patients with LAHPS exhibit various degrees of bleeding, ranging from mild mucocutaneous bleeding to life-threatening intracranial haemorrhage; however, adrenal haemorrhage resulting from LAHPS and its long-term course have only been described rarely [3–5].

Adrenal insufficiency is a potentially life-threatening event, and may result from adrenal haemorrhage [6]. Despite its risk for severe morbidity or mortality, signs and symptoms are subtle and the diagnosis is often delayed [6]. Early recognition of adrenal haemorrhage enables early intervention, and the patient can be managed successfully without endocrine shock after adrenal insufficiency.

We report a rare case of progression of LAHPS into systemic lupus erythematosus (SLE) in a Japanese boy who had severe acute adrenal failure due to bilateral adrenal haemorrhage.

## Case presentation

**Fig. 1:**
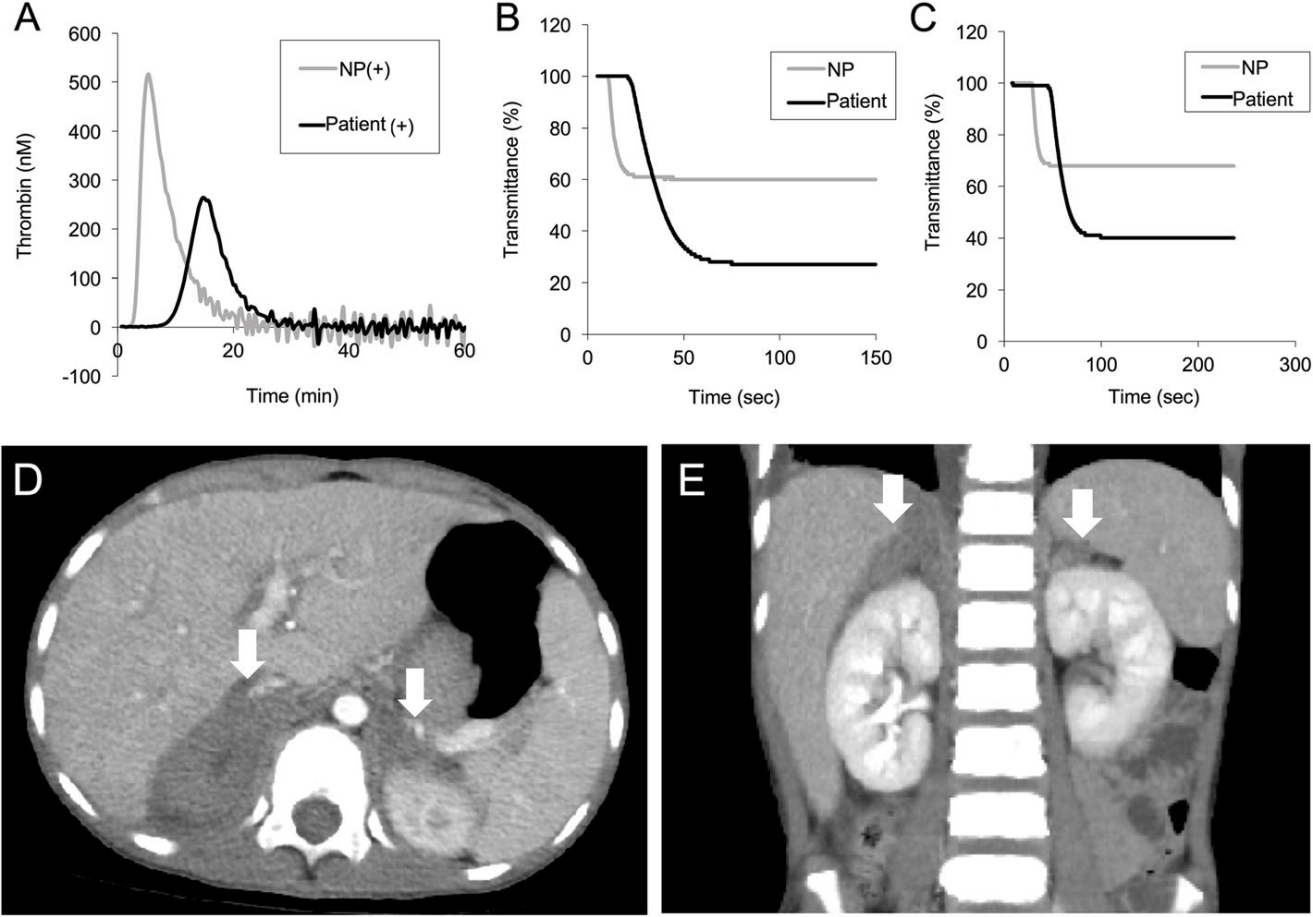
**a** Thrombin generation assay which monitors one-step before fibrin formation. In contrast to a healthy control, the lag time in lupus anticoagulant (LA)-positive serum was delayed. The peak thrombin level in the patient was lower than in the control. **b** and **c** Clot waveform analyses were evaluated by fibrin formation. The clot times in LA-positive serum were significantly prolonged compared to a healthy control. **b**, prothrombin time; **c**, activated partial thromboplastin time. **d** and **e** Contrast abdominal computed tomograms show nodular lesions in the enlarged adrenal glands bilaterally, indicating adrenal haemorrhage (arrows). **d**, Axial; **e**, Coronal

A 9-year-old boy had normal perinatal history, growth, and development. He presented with a fever, abdominal pain, and vomiting, all starting 5 days before admission. Diarrhea and hematochezia were not noted. We examined a stool sample to test for bacterial pathogens, which were not detected. His condition was provisionally diagnosed as acute viral gastroenteritis based on the clinical manifestations. On admission, his vital signs were body temperature 38.0 °C, blood pressure 98/44 mmHg, heart rate 82/min, and respiratory rate 16/min with an O_2_ saturation of 99% on room air. Upper abdominal tenderness was found without abdominal swelling or hepatosplenomegaly. Complete blood count showed white blood cells 9540/μL, haemoglobin 13.9 g/dL, and platelets 140 × 10^3^/μL. Biochemical parameters showed total bilirubin 0.56 mg/dL, aspartate transaminase 58 IU/L, alanine transaminase 47 IU/L, blood urea nitrogen 6.3 mg/dL, creatinine 0.23 mg/dL, sodium 134 mEq/L, potassium 4.1 mEq/L, and C-reactive protein (CRP) 3.7 mg/dL. Coagulation studies revealed prolonged activated partial thromboplastin time (aPTT) of 92.4 s, elevated D-dimer 3.7 μg/mL, LA positivity, and slightly low prothrombin activity 58% (reference range [RR] 75–135%) in combination with immunoglobin M (IgM) class anti-prothrombin antibody of 32.1 AU/mL (RR < 24.0 AU/mL). Immunoglobin G (IgG) class anti-phosphatidylserine/prothrombin antibody was also positive (> 50.0 units, RR < 2.0 units), which is associated with strong LA activity. The patient’s LA-positive plasma was examined using the thrombin generation test and clot waveform analyses (Fig. 1a, b, and c) as previously described [7]. The clotting times in LA-positive plasma were significantly prolonged, compared to a healthy control.

**Fig. 2:**
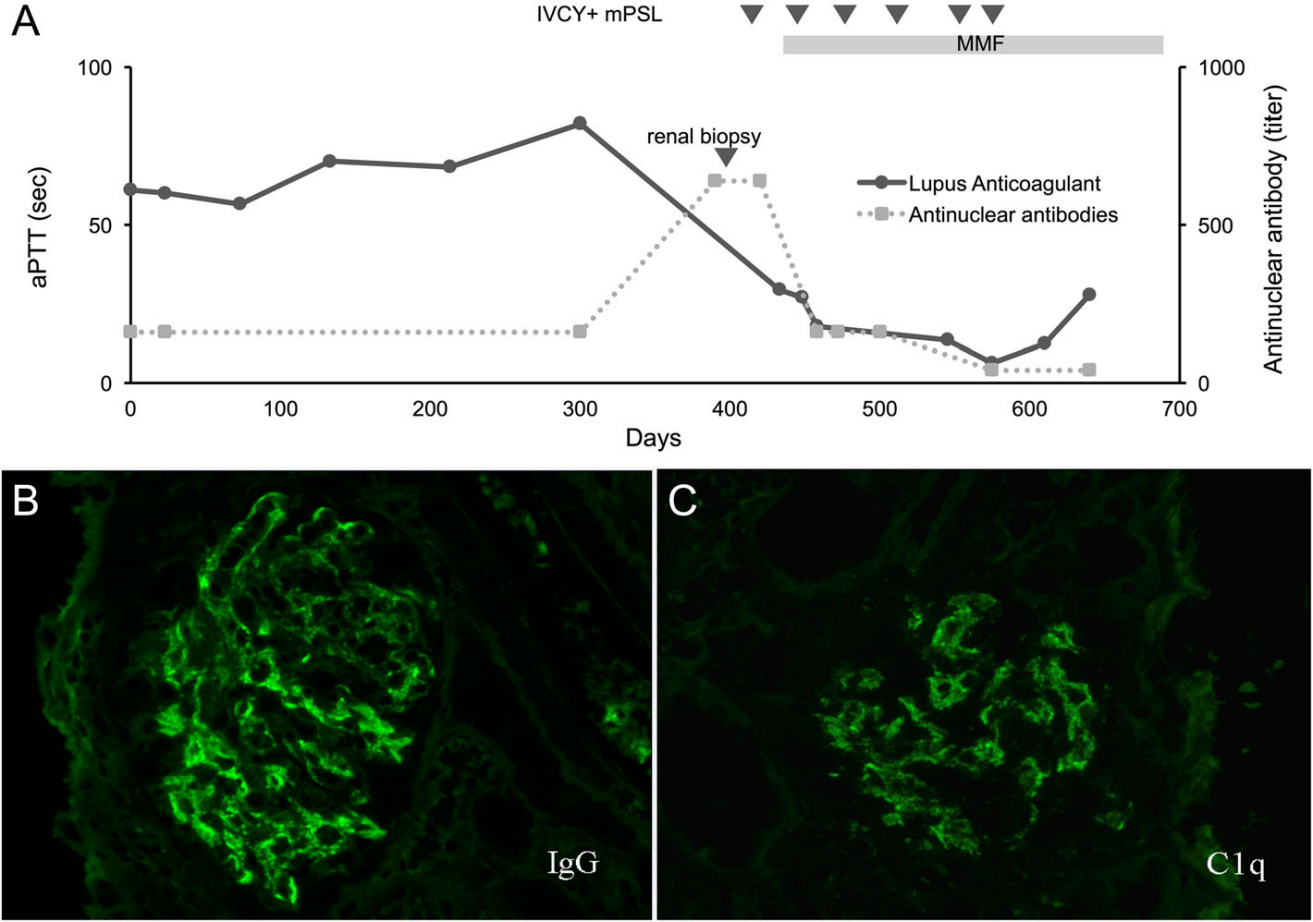
**a** The clinical course of the patient. Lupus anticoagulant remained high, even with hydrocortisone administration. At diagnosis of SLE, high levels of antinuclear antibody are shown. After intravenous pulse treatments with methylprednisolone (30 mg/kg/day) and cyclophosphamide (500 mg/m^2^), a decrease in lupus anticoagulant and antinuclear antibody levels are shown. Abbreviations: IVCY, intravenous cyclophosphamide; mPSL, methylprednisolone; MMF, mycofenolate mofetil; aPTT, activated partial thromboplastin time. **b** and **c** Immunofluorescence micrographs of renal biopsy specimens. **b**, granular IgG deposits in the glomerular capillary walls, in a diffuse and global distribution. **c**, granular deposits of C1q on the glomerular basement membrane

A diagnosis of LAHPS was made. The patient tested positive for anti-nuclear antibody (ANA) titer 1:160, anti-double-stranded DNA (dsDNA) antibody of IgG 22 IU/mL, anticardiolipin antibody of IgG 16 U/mL, and anti-β2-glycoprotein I antibody of IgG > 50 units, but the patient did not fit the Systemic Lupus International Collaborating Clinics (SLICC) 2012 Classification Criteria [8]. A contrast-enhanced computed tomogram revealed nodular lesions in the adrenal glands bilaterally (Fig. 1d and e). The diagnosis of acute adrenal failure due to adrenal haemorrhage was made on the basis of the clinical manifestations, mild hyponatremia (134 mEq/L), high plasma ACTH 1586 pg/mL (RR 7.2–63.3), low plasma cortisol 3.24 μg/dL (RR 6.2–18.0), blood glucose 37 mg/dL, low serum aldosterone 43.1 pg/mL, and relatively elevated plasma renin activity 9.1 ng/mL/hr. Hence, the patient received glucose (0.6 g/kg/dose), hydration (1700 mL/m^2^), and hydrocortisone (50 mg/m^2^/day). Hydrocortisone and fludrocortisone were continued for the adrenal replacement therapy at physiological doses. Repeated coagulation studies still showed positive LA and prolonged aPTT for 12 months (Fig. 2a).

At 10 years of age, he visited our hospital because of gait disturbances and weakness in all extremities after acute upper respiratory viral infection. He had butterfly rash, discoid rash, optic disc swelling, deranged renal function, and showed higher levels of ANA and anti-dsDNA antibody. Renal biopsy resulted in a diagnosis of lupus nephritis (Fig. 2b and c). His condition was diagnosed as SLE on the basis of the SLICC 2012 Classification Criteria [8]. Intravenous pulsed treatments with methylprednisolone (30 mg/kg/day) and cyclophosphamide (500 mg/m^2^) were immediately initiated, followed by mycophenolate mofetil 400 mg/m^2^/day. The patient’s clinical condition improved.

## Discussion and conclusions

The present case provides two clinical messages: 1) early diagnosis and treatment are crucial for a favorable outcome after adrenal failure following adrenal haemorrhage, and 2) we emphasize the importance of careful observation of the patient with autoantibodies, LA and ANA, because autoantibodies precede clinical manifestations of autoimmune diseases, such as SLE [9].

LAHPS is mainly found in children with LA and is accompanied by a decrease in plasma prothrombin activity due to IgM class anti-prothrombin antibody [3, 4]. LAHPS was reported by Bajaj in 1983, and the pathophysiology of LAHPS is thought to be an immune complex with prothrombin antibody, which is rapidly excreted from the blood, resulting in low prothrombin activity [10]. In our case, a decrease in prothrombin activity supports a similar pathophysiology. LAHPS-associated adrenal haemorrhage is rare; however, paediatricians should pay attention to this complication for unexplained or prolonged abdominal pain.

Adrenal failure due to adrenal haemorrhage is rare, but potentially fatal [6, 11]. In childhood, it generally presents with nonspecific signs and symptoms, such as fatigue, malaise, abdominal pain, nausea, and vomiting, without hyperpigmentation. Therefore, diagnosis and treatment may often be delayed. Our case showed prolonged abdominal pain and deranged coagulation, and early recognition of adrenal insufficiency due to adrenal haemorrhage enabled early intervention. The pathophysiology of adrenal gland bleeding remains unclear. The adrenal gland comprises a rich arterial supply with a single vein limiting blood drainage; a thrombosed vein can result in progressive increase in arterial blood pressure [12].

Antibodies promoting thrombosis are known to be associated with autoimmune diseases, such as antiphospholipid syndrome, or to be produced in reaction to an acute infection, particularly in children [13, 14]. However, our patient developed SLE after 1 year, a development described less frequently than the opposite one [9, 15]. Moreover, the patient did not fit the SLICC diagnostic criteria for SLE, nor is haemorrhage, with the partial exception of alveolar haemorrhage, a typical presentation of SLE. However, autoantibodies, including LA and ANA, persisted [16]. The development of autoantibodies has been reported to precede clinical manifestations of autoimmune diseases, such as SLE [9]. Renal dysfunction was also observed when SLE was diagnosed in our case. Therefore, we emphasize that the finding of autoantibodies in a patient with coagulation disorders should be followed by long-term monitoring.

In conclusion, the development of acute adrenal failure due to bilateral adrenal haemorrhage in the context of LAHPS is a rare, but life-threatening event that should be recognized and treated early. Our case stresses the importance of careful observation of the patient with LA and ANA.

## Data Availability

All relevant data were included in this case study.

## Abbreviations

LAHPS: Lupus anticoagulant-hypoprothrombinemia syndrome
LA: Lupus anticoagulant;
SLE: Systemic lupus erythematosus
CRP: C-reactive protein
aPTT: Activated partial thromboplastin time
IgM: Immunoglobin M
IgG: Immunoglobin G
ANA: Anti-nuclear antibody
dsDNA: Double-stranded DNA
SLICC: Systemic lupus international collaborating clinics
